# Vital Pulp Therapy in Permanent Teeth Diagnosed with Symptomatic Irreversible Pulpitis: Reports with Long-Term Controls

**DOI:** 10.1155/2023/2694388

**Published:** 2023-11-23

**Authors:** Abel Teves-Cordova, Luis Coloma Calle, Pierre Mejia Rojas, Juan Goncalves-Pereira, Marco Antonio Hungaro Duarte

**Affiliations:** ^1^Department of Operative Dentistry, Endodontics and Dental Materials, Bauru School of Dentistry, University of São Paulo-USP, Bauru, Brazil; ^2^Faculty of Dentistry of the Catholic University of Cuenca-Azogues, Ecuador; ^3^Department of Endodontics, School of Dentistry, Cayetano Heredia Peruvian University, Lima, Peru; ^4^Department of Endodontics, School of Dentistry, Central University of Venezuela, Caracas, Venezuela

## Abstract

The aim of this study was to describe the treatment of permanent teeth diagnosed with irreversible pulpitis, which can be effectively managed with partial or total pulpotomy. This alternative approach has shown great clinical and radiographic success in the long term compared to traditional pulpectomies. In this series of clinical cases, all the teeth exhibited symptoms of intense pain upon exposure to cold and at night. The clinical examination revealed extensive caries, while radiographic imaging showed radiolucent lesions in contact with the pulp chamber, indicating symptomatic irreversible pulpitis. The chosen treatment approach was either partial or total pulpotomy. The tooth was anesthetized, and the operative field was isolated and disinfected. After removing caries with a sterile round drill, the area was rinsed with sodium hypochlorite. In some cases, a portion of the pulp tissue was removed, while in others, the entire tissue of the pulp chamber was extracted using diamond burs. Hemostasis was achieved by applying sterile cotton pellets for 2 to 6 minutes. Following that, the tissue exhibited no signs of bleeding. Bioceramic cements were used, and the tooth was definitively restored. Periodic follow-up examinations were conducted, consistently showing positive pulp responses and no evidence of periradicular radiolucent lesions on radiographs.

## 1. Introduction

One of the causes of pulp inflammation and infection is dental caries, which initiates an inflammatory response process and eventually leads to pulp necrosis; the dental pulp serves important functions such as formation, nutrition, protection, and repair. When it becomes infected by bacteria, it manifests signs and symptoms of reversible pulpitis. Without treatment, the disease progresses to an irreversible state, leading to consequences such as infections, tooth loss, pain, and a decline in self-confidence [1]. Currently, the standard treatment for irreversible pulpitis is root canal treatment; this procedure involves the complete removal of inflamed and infected pulp tissue to prevent the development of apical periodontitis and to preserve the tooth within the oral cavity [2].

Nowadays, advancements in materials and research in histology and microbiology have led to improved conservative protocols for dental pulp treatment [3]. Consequently, endodontic societies are proposing new guidelines for managing situations involving deep caries, extremely deep caries, and irreversible pulpitis [4]. However, accurate diagnosis of the pulp condition is crucial in determining the appropriate therapy [5]. Vital pulp therapy involves various approaches, including indirect and direct pulp capping, as well as partial or total pulpotomy. The primary goal is to maintain pulp vitality and provide the most effective and conservative treatment that is also affordable for the patient [6]. By utilizing vital pulp therapy, the risk of potential complications during conventional root canal treatment, such as tooth perforation, chlorine extravasation, or instrument fractures, can be minimized [7, 8].

Partial and total pulpotomies are not new treatments. Initially, it was recommended for treating permanent teeth with immature apices, where complete root formation is necessary. However, new clinical trial studies provide evidence that it can be an alternative treatment for cases involving mature permanent teeth with a fully formed apex and irreversible pulpitis [9, 10].

A correct protocol for isolation, asepsis, and clinical evaluation during the intraoperative procedure for bleeding and hemostasis allows for greater success in treatment [11]. Sodium hypochlorite, with a concentration of 1 to 5.25%, can be a good alternative for irrigating the remaining pulp tissue due to its antibacterial properties, and randomized controlled trials have also shown a decrease in postoperative pain with the use of this substance [12]. Another important factor to consider is the choice of capping material. Historically, calcium hydroxide was the most commonly used material in vital pulp therapy. However, recent clinical studies have shown that MTA and calcium silicate-based cements yield better results when treating deep carious lesions with exposed pulps, leading to favorable outcomes. Additionally, calcium silicate materials possess properties of cellular biocompatibility [13, 14] and bactericidal effects [15].

Follow-up studies have shown that vital pulp therapy (VPT) has a high success rate when combined with proper restoration, effectively preventing bacterial infiltration and contamination in the oral cavity [16]. It is considered a less invasive, cost-effective, and pulp-preserving alternative. However, there is a lack of long-term follow-up studies that could further support the existing research. Two published studies, a systematic review and a meta-analysis of randomized clinical trials, suggest the need for studies with extended follow-up periods [17, 18]. Therefore, the objective of this paper is to present a case series comprising 8 clinical cases of permanent teeth diagnosed with irreversible pulpitis and managed with partial pulpotomy and total pulpotomy, with follow-up assessments conducted for a minimum of 2 years.

## 2. Case Description and Results

**Figure 1:**
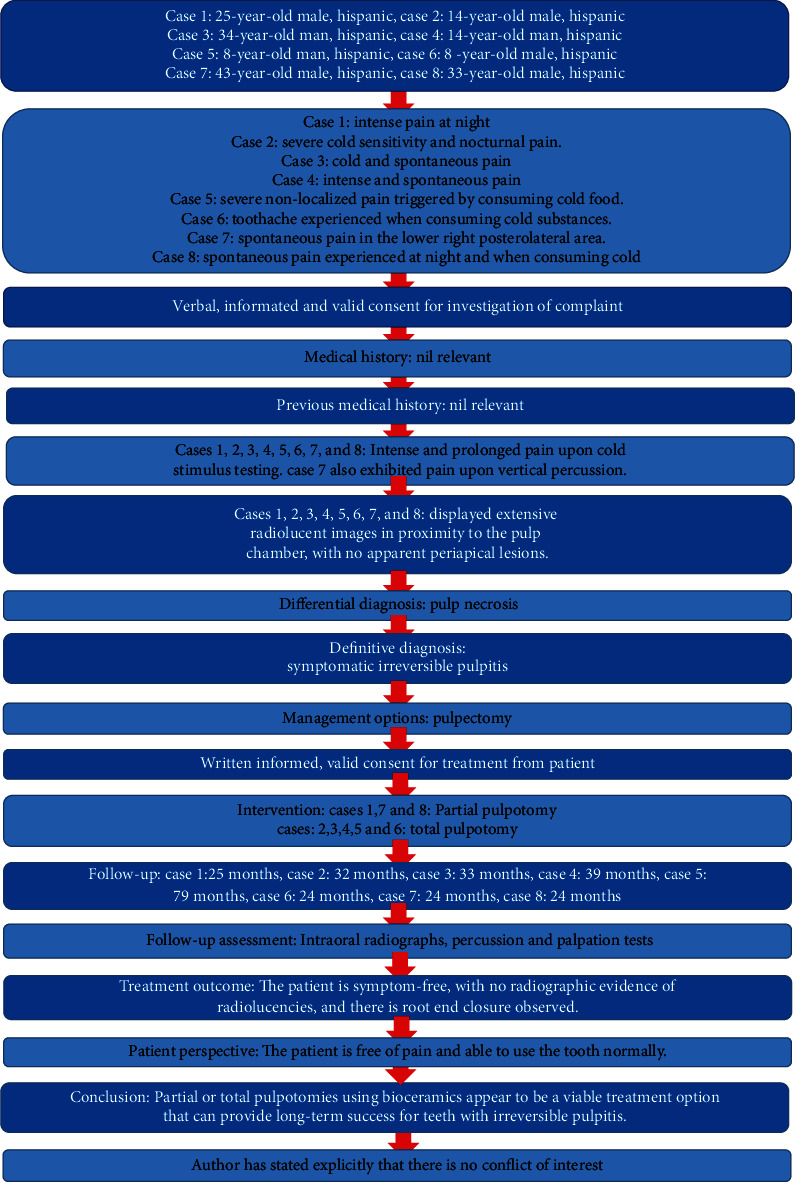
This flow chart from PRICE 2020 guidelines for reporting case reports in endodontics (http://pride-endodonticguidelines.org/price/).

This case report has been written according to the Preferred Reporting Items for Case Reports in Endodontics (PRICE) 2020 guidelines [19] (Figure 1).

**Figure 2:**
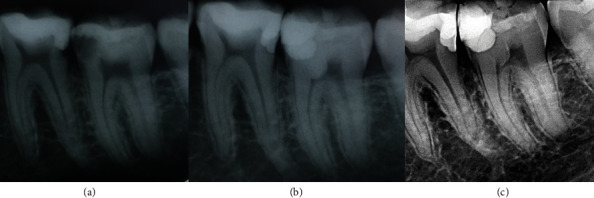
Permanent tooth 37. The image (a) shows diagnostic X-ray, image (b) immediate postoperative, and image (c) control at 25 months.

**Figure 3:**
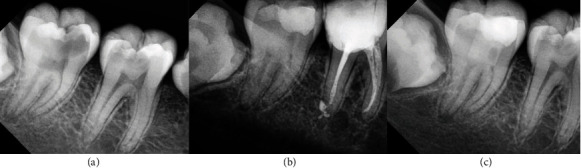
Permanent tooth 47. The image (a) shows diagnostic X-ray, image (b) immediate postoperative, and image (c) control at 32 months.

**Figure 4:**
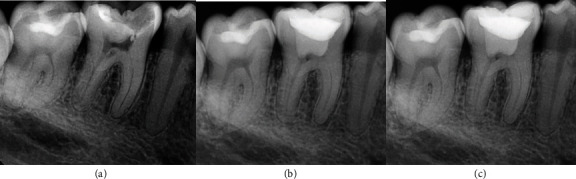
Permanent tooth 46. The image (a) shows diagnostic X-ray, image (b) immediate postoperative, and image (c) control at 33 months.

**Figure 5:**
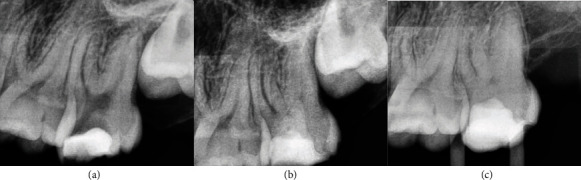
Permanent tooth 27. The image (a) shows diagnostic X-ray, image (b) immediate postoperative, and image (c) control at 39 months.

**Figure 6:**
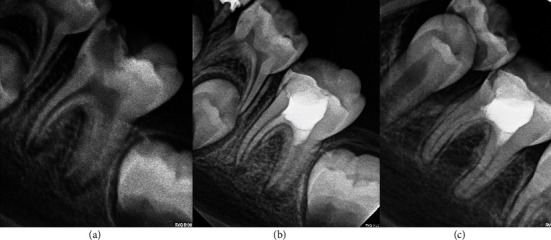
Permanent tooth 36. The image (a) shows diagnostic X-ray, image (b) immediate postoperative, and image (c) control at 79 months.

**Figure 7:**
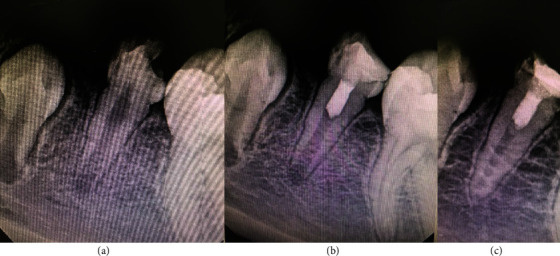
Permanent tooth 35. The image (a) shows diagnostic X-ray, image (b) immediate postoperative, and image (c) control at 24 months.

**Figure 8:**
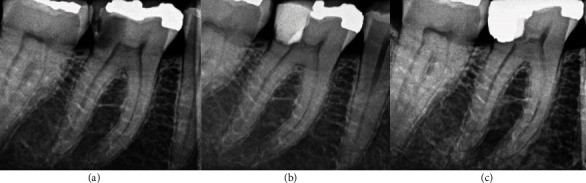
Permanent tooth 46. The image (a) shows diagnostic X-ray, image (b) immediate postoperative, and image (c) control at 24 months.

**Figure 9:**
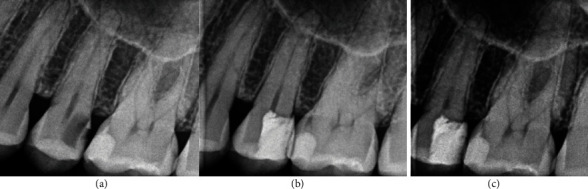
Permanent tooth 25. The image (a) shows diagnostic X-ray, image (b) immediate postoperative, and image (c) control at 24 months.

Eight Hispanic patients who experienced intense and spontaneous pain requiring immediate treatment were evaluated. Four different clinicians administered all treatments. During the clinical examination, they exhibited signs and symptoms of pulp inflammation as described in Table 1. Additionally, the radiographic examination revealed radiolucent images in direct contact with the pulp chamber in some cases. Only one case (patient 4) had a temporary restoration, which appeared as a radiopaque image in contact with the pulp chamber on the radiograph. The permanent teeth had mature apices, except for patients 5 and 6, who had immature apices. The diagnoses for all cases were symptomatic irreversible pulpitis with normal periapical tissues, except for patient 7, who had a symptomatic apical periodontitis. The chosen treatment for all cases was either partial or total pulpotomy. Before signing the informed consent form, the patients were informed about the benefits and disadvantages of the treatment compared to other options. Once informed consent was obtained, both for treatment and for the publication of clinical cases in journals, the treatments were carried out (Figures 2–9).

The teeth were anesthetized with different anesthetics (Table 1) under absolute isolation and disinfection of the operative field. Nonselective removal of caries was performed in the following manner: the caries were excavated using a large sterile round diamond drill, starting from the outermost area and progressing towards the inner part. Once all the carious lesion was removed, a new drill was used to make contact with the pulp tissue. This procedure was carried out at a high speed with water coolant. After the pulp was exposed, the opening was refined using a sterile conical trunk diamond drill. The presence of pulp hemorrhage upon entering the pulp chamber confirmed the clinical diagnosis of a vital pulp. The exposed pulp was carefully removed using a large sterile round diamond cutter in a high speed, while being cooled with water coolant.

In the evaluation of the clinicians, it was considered that all the whitish and avascular tissue had been completely removed. The inflamed tissue was deemed irreversibly damaged due to the abundant bleeding it exhibited. The pulp tissue that was not removed should have a bleeding time between 2 and 6 minutes and show no morphological changes. If the bleeding is not stopped, root canal treatment will be necessary. In our cases, everyone had an adequate hemostasis time.

**Figure 10:**
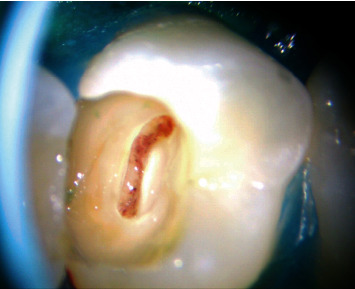
The image shows the hemostasis of pulp tissue under the Vasconcelos microscope at 20x magnification.

The pulp tissue was completely removed up to the level of the root canal orifices (Figure 10) in teeth undergoing total pulpotomy. In two patients (patients 1 and 7), the tissue was excised to a depth of 3-5 mm from the entire coronal pulp, representing a partial pulpotomy. Hemostasis was achieved in the teeth following the pulpotomy procedure by placing sterile cotton pellets in the pulp wound for a duration ranging from 2 to 6 minutes. The cavity was rinsed with 1 to 5.25% sodium hypochlorite in most cases, while the saline solution was used in two cases (Table 1).

Next, bioceramic cement was used as pulp capping material according to the manufacturer's instructions. After pulp capping, the teeth were restored with glass ionomer and light-curing resin. Only one case was restored with amalgam (Figures 2–9).

The immediate post-treatment evaluation showed that most cases did not have postoperative pain. Only the first patient experienced pain for 1 week. Periodic follow-up examinations were conducted, evaluating pulp vitality, palpation, and percussion pain. All teeth were asymptomatic and responded normally to the thermal test. There was no pain upon percussion or palpation, and radiographically, none of the teeth showed evidence of periapical radiolucent lesions. Additionally, no obliterations of the root canal were observed. In the unlikely event that they had occurred, an urgent canal treatment (pulpectomy) would have been planned. Two cases with immature apices exhibited complete formation of the root apices (Table 1).

## 3. Discussion

The success in treating pulp inflammation and infection lies in eliminating the underlying cause that either releases products or directly contacts the pulp tissue, leading to inflammation [20]. When bacteria come into contact with the tissue, it causes pain in response to thermal stimuli, osmotic solutions, and even spontaneous pain [21].

The treatment of choice for this disease for a long time was pulpectomy, but now with new advances in microbiology, histology, and pulp capping materials, treatments are more conservative in preserving pulp vitality. The principal options now are partial and total pulpotomies. Currently, many studies have evaluated conservative treatment as an option and have demonstrated very promising results, showing high clinical and radiographic success [22].

The use of aseptic isolation, magnification, and bioceramic cements [23–26] has favored an increase in success rates. When compared with calcium hydroxide [27, 28], which has been widely used for many years, bioceramics as pulp capping material have shown a higher percentage of success.

The cases presented demonstrated that vital pulp therapy with pulpotomies can be a good option in permanent teeth with open and closed apexes that present symptomatic irreversible pulpitis. The development of a proper diagnosis, along with the application of appropriate protocols described by other authors [6], has shown successful outcomes in all cases where pulpotomy was performed using various bioceramic cements [23, 28–30].

Bioceramic cements produce portlandite, which is calcium hydroxide, during hydration and setting. Portlandite is responsible for the bioactivity and biocompatibility of the material. Additionally, its low solubility and ability to induce dental pulp proliferation and tissue formation are properties that make this material favorable for use as a pulp capping material.

In our cases, all teeth achieved hemostasis between 2 and 6 minutes. Studies by Lin et al. have shown that the appropriate time for hemostasis should be between 8 and 15 minutes. If bleeding persists beyond this time frame, one should consider performing a pulpectomy [3]. Another study by Ricucci et al. found that if bleeding cannot be stopped within a reasonable time, the treatment procedure should be modified; this may involve changing from a partial pulpotomy to a complete pulpotomy, or from a complete pulpotomy to a pulpectomy; the suggested time for intervention in their study is 2 minutes [31]. Finally, a current study by Santos et al. conducted an in vivo histological study in an animal model and found that the time of hemostasis does not significantly influence the success of pulpotomies in permanent teeth [6]. The divergent results from these studies are still subject to debate, and further research is needed to gain a clearer understanding.

The cleaning and disinfection of pulp exposure during the pulpotomy procedure is as important as the pulp capping cement. The cases presented in this study were irrigated with sodium hypochlorite in different concentrations ranging from 1% to 5.25%. Munir et al. found in a systematic review that there is a tendency to use sodium hypochlorite in various concentrations in vital pulp therapy, but more studies are needed [32]. Only two cases in this study utilized saline solution for irrigation, and they showed success, consistent with a previous study by Brizuela et al., which found positive results with saline solution irrigation. However, irrigating with sodium hypochlorite has the advantage of maintaining an aseptic environment, making it the preferred cleaning solution option [33].

The teeth were restored immediately in most cases. Only in two cases, the restoration was performed provisionally with glass ionomer, and after a week, it was definitively restored. A previous study showed that glass ionomer was a good material as it prevented bacterial filtration even after one month [34].

The follow-up of the cases presented in this study was a minimum of 2 years, showing that the treatments were effective in the long term. Currently, most studies have a follow-up period of 1 or 2 years, and there are few studies with a longer follow-up period that demonstrate a high percentage of success [35]. In the radiographic evaluation, the formation of the dentin barrier (Figures 8 and 9), presence of pulp vitality [25], and absence of periapical lesions were observed. The closure of the apices was also observed in immature permanent teeth. Recent evidence does not provide many documented follow-up cases of vital pulp therapy performed with bioceramics and with long-term success. Therefore, it is imperative to conduct studies that include controls over a longer period, as demonstrated in this paper.

## 4. Conclusion

Vital pulp therapy with partial or total pulpotomies using bioceramics under aseptic conditions is a viable treatment that will have long-term success in permanent teeth with irreversible pulpitis when indicated correctly.

## Figures and Tables

**Table 1:** Description of the most important data for each patient's treatment.

Patient	Tooth	Tests clinical	Apex	Type of anesthesia	Irrigant	Hemostasis time	Pulp capping material	Reconstruction
Patient 1, 25-year-old male (Figure 2)	FDI 37	Cold: positive Heat: negative Palpation: negative Percussion: negative	Closed	Mepivacaine 2% with levonordefrin 1 : 20,000	1% sodium hypochlorite	3 minutes	Biodentine	Immediate, glass ionomer, and resin

Patient 2, 14-year-old male (Figure 3)	FDI 47	Cold: positive Heat: negative Palpation: negative Percussion: negative	Closed	Articaine 4% with adrenaline 1/100,000	Sodium hypochlorite 2.25%	2-6 minutes	Biodentine	Immediate, glass ionomer, and resin

Patient 3, 34-year-old man (Figure 4)	FDI 46	Cold: positive Heat: negative Palpation: negative Percussion: negative	Closed	Articaine 4% with adrenaline 1/100,000	Sodium hypochlorite 2.25%	2-6 minutes	Biodentine	Immediate, glass ionomer, and resin

Patient 4, 14-year-old man (Figure 5)	FDI 27	Cold: positive Calor: negativo Palpation: negative Percussion: negative	Closed	Articaine 4% with adrenaline 1/100,000	Sodium hypochlorite 2.25%	2-6 minutes	Biodentine	Immediate, glass ionomer, and resin

Patient 5, 8-year-old man (Figure 6)	FDI 36	Cold: positive Heat: negative Palpation: negative Percussion: negative	Open	Lidocaine 2% with epinephrine 1 : 80000	Saline solution	2-6 minutes	MTA Angelus	Immediate, glass ionomer, and resin

Patient 6, 8-year-old male (Figure 7)	FDI 35	Cold: positive Heat: negative Palpation: negative Percussion: negative	Open	Lidocaine 2% with epinephrine 1 : 80000	Saline solution	2-6 minutes	NeoMTA2	Immediate, glass ionomer, and resin

Patient 7, 43-year-old male (Figure 8)	FDI 46	Cold: intense pain Heat: negative Palpation: negative Vertical percussion: positive	Closed	2% lidocaine with epinephrine 1 : 100,000.	Sodium hypochlorite 5.25%	5 minutes	EndoSequence BC RRM	After one week, glass ionomer and amalgam

Patient 8, 33-year-old male (Figure 9)	FDI 25	Cold: intense pain Heat: negative Palpation: negative Vertical percussion: negative	Closed	2% lidocaine with epinephrine 1 : 100,000	Sodium hypochlorite 5.25%	5 minutes	EndoSequence BC RRM	After one week, glass ionomer and resin

## Data Availability

Data is available on request from the authors.
